# Understanding why low-risk patients accept vaccines: a socio-behavioral approach

**DOI:** 10.1186/s13104-015-1816-2

**Published:** 2015-12-23

**Authors:** Timothy L. Wiemken, Ruth M. Carrico, Robert R. Kelley, Laura E. Binford, Paula Peyrani, Kimbal D. Ford, Verna Welch, Julio A. Ramirez

**Affiliations:** Department of Medicine, Division of Infectious Diseases, University of Louisville School of Medicine, Louisville, KY 40202 USA; Pfizer Inc., Collegeville, PA USA; Computational Epidemiology Unit, University of Louisville Division of Infectious Diseases, Global Health Research Organization, 501 E Broadway Suite 140B, Louisville, KY 40202 USA

**Keywords:** Immunization, Theory of Planned Behavior, Behavioral intention, Vaccine acceptance, Instrumental variable

## Abstract

**Background:**

Vaccines are one of the most important public health interventions. Understanding factors associated with vaccine acceptance is critical. The objectives of this study were to evaluate the impact of the three constructs of the Theory of Planned Behavior (TPB) on the intention to be vaccinated among healthy individuals being seen for pre-travel care, and to evaluate if behavioral intention was associated with vaccine acceptance.

**Methods:**

We surveyed individuals seeking vaccination at the University of Louisville Vaccine and International Health and Travel Clinic. Linear and two stage least squares regression models were used to define the associations between constructs of the TPB and the intention to be vaccinated, as well as the association between the intention to be vaccinated and vaccine acceptance.

**Results:**

A total of 183 individuals were included in the analysis. None of the constructs of the TPB were associated with intention to be vaccinated. Behavioral intention was not associated with vaccination acceptance.

**Conclusions:**

This study suggests that the TPB does not predict the intention to get vaccinated among individuals attending our Vaccine and International Health and Travel Clinic. It will be critical to define better predictors of vaccine uptake in healthy, low-risk individuals to increase vaccine acceptance.

## Background

Vaccines are one of the most important and successful public health interventions in human history. The growing anti-vaccination movement has resulted in reductions in immunization rates and substantial resurgences in vaccine preventable diseases with associated increases in morbidity and mortality [1]. However, the anti-vaccination movement is largely associated with childhood vaccinations and/or vaccinations in high-risk individuals. It is critical to better understand what factors are associated with healthy people accepting vaccination in order to reduce the burden of illness associated with these diseases. Currently, there are few evaluations of vaccine acceptance and uptake among healthy, low-risk individuals.

The development of behavioral theories has greatly facilitated our understanding of the health behavior of the community through allowing systematic approaches to behavioral research [2]. One such theory, the Theory of Planned Behavior (TPB), has been used to identify factors associated with the intention to perform a behavior, including being immunized. According to this theory, a health behavior can be predicted based on the individuals intention to perform the behavior [3, 4]. There are three major constructs theorized to predict the outcome of the model, “behavioral intention”. These three constructs include: (1) attitude, (2) subjective norms, and (3) perceived behavioral control. Each of these constructs can be measured using previously validated approaches [5], understanding that each construct is the perception of the individual under study and not necessarily what is actually occurring. Regardless, the published data regarding vaccines using this theory has been limited to individuals in high-risk groups obtaining vaccinations for Hepatitis B [6, 7] or influenza vaccine [8], or healthy young females obtaining Human Papilloma Virus vaccine [9–12].

Outside of these applications of the TPB, factors influencing the intention to be vaccinated by otherwise healthy individuals in low-risk groups are not well understood.

The objectives of this study were to evaluate the impact of the three constructs of the TPB on the intention to be vaccinated in healthy individuals being seen for care at a Vaccine and International Travel Clinic, and to evaluate if behavioral intention was associated with actual vaccine acceptance.

## Methods

### Study design and setting

This was a cross-sectional study enrolling individuals at the University of Louisville Vaccine and International Health and Travel Clinics in Louisville, KY from November 2013 to July 2014. All unique individuals with a home address in Louisville being seen for vaccine reasons were invited to participate. Persons requiring only booster vaccines were excluded, as were patients in whom a particular vaccine was required for their intended travel (e.g. yellow fever). The survey was provided to each individual to complete on his/her own via a tablet computer prior to being advised by the healthcare provider. No incentives were provided to participants. The Clinics offer, on a case-by-case basis, vaccines based on recommendations from the Centers for Disease Control and Prevention Advisory Committee on Immunization Practices. All vaccines considered appropriate for subjects are recommended equally. After a full evaluation, subjects are offered an array of vaccines based on their personal medical history and epidemiologic future. This includes vaccines related to their travel, which are recommended, and also vaccines for which they may need based on their medical history (e.g. pneumococcal vaccine or Tdap). The vaccines offered included: Hepatitis A and B, Influenza (injectable, nasal mist, or intradermal), Japanese encephalitis, measles/mumps/rubella (MMR), meningococcal, polio, pneumococcal, rabies, tetanus/diphtheria/pertussis (Tdap), typhoid (injectable or oral), varicella, and zoster. The University of Louisville Human Subjects Protection Program Office approved this study prior to any data collection (Protocol Number 12.0470). Consent was obtained upon agreeing to complete the survey.

### Theoretical rationale and survey

The Theory of Planned Behavior (TPB) was used as a theoretical framework for the present study. The TPB has been used to evaluate individuals’ intention to get vaccinated and therefore we were able to pull from this body of evidence for our questionnaire development [6–12]. The TPB posits that a person’s intention to perform a behavior is the biggest driver for actually performing the behavior. Behavioral intention can be measured via three major constructs (1) Attitudes, defined as a person’s behavioral beliefs and evaluation of those beliefs, (2) Subjective Norms, defined as normative beliefs and motivation to comply with those beliefs, and (3) Perceived Behavioral Control, defined as control beliefs and the person’s perceived power over the behavior. In short, Attitudes are related to the person’s inner feelings and motivations regarding a behavior; Subjective Norms are equivalent to perceived peer pressure and how strongly influenced the person is by peers; and Perceived Behavioral Control is related to how much control the person perceives they have over the behavior.

Our survey included 26 items related to the TPB as well as some basic demographic questions. All TBP questions were graded on a 7-point scale, and were coded −3 (strongly disagree) to +3 (strongly agree), with zero being neutral. The survey is available on our website (http://globalhealth.center/vtc/survey.pdf). For each individual completing the survey, whether or not each offered vaccine was actually accepted and provided was also tracked. These data were used to calculate a composite “percent acceptance” of all offered vaccines for each individual.

### Quality control/data management plan

Data were collected first person on a mobile tablet computer. All data were stored in a clinical data management system (REDCap) on secured servers maintained by the University of Louisville Clinical and Translational Research Support Unit (CTRSU) (ctrsu.net). Quality control and data management were performed by members of the CTRSU.

### Statistical analysis

Although our survey instrument was based on items used by other investigators in similar studies, we evaluated the internal validity of the items prior to our main analysis. First, the internal validity of items was evaluated using Cronbach’s Alpha statistics. Items with Cronbach’s Alpha value of less than 0.6 were examined via boxplots and descriptive statistics to identify items that may not have fit with the other answers. Items that were deemed to not be valid were removed and Cronbach’s Alphas were recalculated. If the statistic decreased, the item was added back to the analysis. Next, we used exploratory factor analysis to further clarify the most appropriate survey items to include as measurements for the main TPB constructs. Items that loaded poorly (eigenvalue <0.55) with other items theoretically bound to the same construct were evaluated via boxplots and descriptive statistics. Items deemed to not be valid were removed and Cronbach’s Alphas were recalculated. If this statistic decreased, the items were added back. After the instrument had passed all internal validity checks, medians of all items in each construct were calculated. To determine the impact of each construct on the intention to be vaccinated, we used linear regression analysis. The outcome of the model was the median of the items related to the intention to perform the behavior. The three TPB variables included in the model were comprised of the median of all of the survey items used to measure the respective construct. Our statistical approach included traditional linear regression modeling, adjusting for the following confounding variables: age ≥56 years, sex, and having a baccalaureate degree or higher. We also considered the construct “perceived behavioral control” to have an endogenous relationship with the outcome “behavioral intention” since we considered perceived behavioral control to predict behavioral intention, but also behavioral intention to predict perceived behavioral control. This rationale stemmed from the precursor theory to the TPB, the Theory of Reasoned Action, which is identical to the TPB but without the construct perceived behavioral control. Since intention can theoretically be in place without perceived behavioral control based on the Theory of Reasoned Action, it is likely that an individual with the intention to perform a behavior may influence her or his own perceived control over the situation. Because of this endogenous relationship between a predictor variable and the outcome variable, we also chose to construct two stage least squares regression models using an instrumental variable, the geographic area of Louisville of primary residence (west, downtown/central, south, east). These regions were based on income, crime, and other demographic data obtained from local sources. Geographic instruments have been shown to be successfully utilized in previous studies using this statistical approach [14, 15]. Finally, the percent acceptance for each individual, as described above, was used as the outcome variable in a linear regression model to evaluate the correlation between the median response for the behavioral intention questions on the survey with actual acceptance and provision of all offered vaccines. Regression diagnostics were conducted on all models to ensure assumptions were met, including evaluation of Q–Q plots, Cooks’ D, homoscedasticity, and studentized residuals. Multicollinearity was assessed using variance inflation factor statistics.

## Results

A total of 183 individuals were included in the analysis. Of the 26 TPB items on the survey instrument, six items were removed due to concerns with internal validity via Cronbach’s Alpha or factor analysis. A total of 76 (41.5 %) of respondents were male, 52 (28.4 %) were of age 56 or older, 9 (5 %) were African American, and 140 (76.9 %) reported being educated at the baccalaureate level or higher. Table 1 describes the baseline characteristics of all 183 subjects. Table 2 depicts the major constructs of the TPB with their respective Cronbach’s Alpha values after removal of problematic items. Results of the regression analyses can be found in Tables 3 (linear) and 4 (2-stage least squares). None of the constructs of the TPB were associated with the respondent’s intention to be vaccinated. In both models, having a baccalaureate degree or higher was significantly associated with an increase in intention to be vaccinated (p = 0.002, 0.006, respectively). Behavioral intention was also not associated with acceptance and provision of vaccine (β = 3.41, Standard Error = 1.94, t = 1.76, p = 0.08).

**Table 1:** Baseline characteristics of 183 subjects

Variable	n (%)
Age	
<18 years	4 (2.2)
18–25 years	30 (16.4)
26–35 years	44 (24.0)
36–45 years	24 (13.1)
46–55 years	29 (15.8)
56–65 years	36 (19.7)
≥65 years	16 (8.7)
Male sex	76 (41.5)
Race	
White/caucasian	160 (88.4)
Black/African American	9 (5.0)
Asian	7 (3.9)
Other	5 (2.8)
Currently married	90 (49.5)
Currently has health insurance	167 (92.8)
Currently employed	138 (75.8)
Annual income	
0–$25,000	12 (7.0)
25,001–$50,000	27 (15.7)
$50,001–$75,000	17 (9.9)
$75,001–$100,000	31 (18.0)
>$100,000	57 (33.1)
Refuse	28 (16.3)
Highest education	
Less than high school diploma	5 (2.7)
High school diploma/GED	29 (15.9)
Associate degree	8 (4.4)
Bachelor degree	67 (36.8)
Master degree	49 (26.9)
Doctoral degree	15 (8.2)
Other professional degree	9 (4.9)
Religious affiliation guides vaccination decisions	21 (12.1)
Attitudes score, median (IQR)	2.5 (0.83)
Normative beliefs score, median (IQR)	1.6 (0.86)
Perceived behavioral control score, median (IQR)	1.5 (1.0)
Percent acceptance of offered vaccine, median (IQR)	66.7 (60)

**Table 2:** Final Cronbach’s Alpha scores for internal validity of items in each construct of the Theory of Planned Behavior

Construct	Number of survey items	Cronbach’s Alpha
Attitudes	6	0.95
Subjective norms	7	0.70
Perceived behavioral control	4	0.81
Behavioral intention	3	0.70

**Table 3:** Results of the adjusted ordinary least squares regression

Variable	β	Standard error	t statistic	P value
Intercept	−0.47	0.51	−0.93	0.355
Attitudes	0.12	0.09	1.37	0.173
Subjective norms	0.21	0.12	1.84	0.067
Perceived behavioral control	0.13	0.19	0.70	0.484
Male sex	−0.06	0.19	−0.32	0.751
Age ≥56 years	0.23	0.20	1.10	0.272
Baccalaureate degree or higher	0.69	0.22	3.14	0.002

**Table 4:** Results of the adjusted 2 stage least squares regression using the geographical region of Jefferson County, Kentucky as an instrumental variable

Variable	β	Standard error	t statistic	P value
Intercept	0.14	2.15	0.07	0.947
Attitudes	0.13	0.09	1.39	0.166
Subjective norms	0.25	0.18	1.42	0.157
Perceived behavioral control	−0.14	0.95	−0.15	0.881
Male sex	0.21	0.21	0.97	0.332
Age ≥56 years	−0.07	0.19	−0.38	0.708
Baccalaureate degree or higher	0.74	0.26	2.80	0.006

## Discussion

This study suggests that the constructs of the TPB may not be useful in predicting the intention to get vaccinated among individuals attending a clinic that specializes in vaccines and international travel. However, increased education appears to be an important factor associated with substantial increases in the intention to be vaccinated. Interestingly, behavioral intention was not found to be associated with actual acceptance of the vaccine.

Since the TPB is focused on individual perceptions, it is possible that interpersonal (e.g. internal perceptions), community, organizational, or policy-level factors may play a more important role in a healthy individual’s intention to become vaccinated for travel-related reasons. This may explain why we were not able to define a significant association between the intrapersonal constructs of the TPB and behavioral intention.

Other studies have reported conflicting evidence between the association of the three constructs of the TPB and behavioral intention with respect to vaccination decisions. For example, significant associations between parents’ intentions to vaccinate their children and all three of the constructs of the TPB have been previously documented [13]. Another study reported no association between the constructs of the TPB and injection drug users’ intention to be vaccinated with the Hepatitis B vaccine [6]. More importantly, some data suggest that even if the constructs are associated with intention, intention may not actually be associated with actually obtaining the vaccine [12], a finding we also documented. Our data suggest that it is still unclear as to this theory’s use in predicting vaccination decisions.

Our study has several limitations. First, it is possible that the results were biased due to the self-report nature of our study. Second, since individuals attending our clinic are typically attending because they would like to be healthy on their travels, they may be more likely to intend to be vaccinated or accept a vaccine if offered. This may represent some selection bias. The generalizability of our study is limited to individuals with similar demographic characteristics. Our data indicate that many of the patients attending our Vaccine and International Health and Travel Clinics represent a very specific demographic and socioeconomic group who may already be motivated to accept vaccines. It should be kept in mind that these individuals are seeking care, potentially seeking vaccines, and likely have a much higher probability of accepting vaccines offered to them than the general population. Furthermore, it is possible that different factors outside of one’s perceptions influence behavioral intention among different populations who were not included in our sample. Another important limitation is that our sample size limited our array of possible analytical techniques. It is possible that path analysis or structural equation modeling may have provided different results. Response bias is always a possibility in self-administered surveys due to misunderstanding of items, negative/positive wording of alternate questions, or other reasons. Although we were assiduous in our efforts to ensure the validity of our survey instrument, the internal validity assessment did find some issues with certain items, which might suggest potential bias. The main strength of our study is that we collected data from consecutive individuals in our clinic over a 9 month period and we conducted an instrumental variables analysis to account for the endogenous relationship between behavioral intention and perceived behavioral control.

## Conclusions

In conclusion, we were not able to document any association between the constructs of the TPB on the intention to be vaccinated when attending a Vaccine and International Health and Travel Clinic. It will be critical to define more reliable predictors of vaccine uptake in healthy, low-risk individuals to increase vaccine acceptance and to prevent future outbreaks of vaccine preventable diseases.
